# Editorial: Use of Ultrasound in Diagnosis and Treatment of Peripheral Nerve Entrapment Syndrome

**DOI:** 10.3389/fneur.2019.01348

**Published:** 2020-01-09

**Authors:** Ke-Vin Chang, Sang Beom Kim

**Affiliations:** ^1^Department of Physical Medicine and Rehabilitation, National Taiwan University Hospital, Bei-Hu Branch and National Taiwan University College of Medicine, Taipei, Taiwan; ^2^Department of Neurology, College of Medicine, Kyung Hee University Hospital at Gangdong, Kyung Hee University, Seoul, South Korea

**Keywords:** ultrasound, peripheral nerve, entrapment syndrome, neuropathic pain syndromes, diagnosis and treatment

In the recent years, ultrasound has revolutionized the assessment and intervention for musculoskeletal pain. It provides real time imaging of the muscles (1), tendons (2), bones and nerves (3), which enables the clinicians to make a prompt diagnosis. One of the most significant progress is the use of ultrasound in evaluation of nerve entrapment disorders. High resolution ultrasound facilitates visualization of individual nerve fascicles whose sizes and configuration may alter in different peripheral nerve diseases. Therefore, in order to raise the awareness of how useful ultrasound can be for the diagnosis of nerve entrapment syndromes, we initiated a special issue probing high-quality original research and review articles in this emerging field.

In this special issue, we successfully collected five intriguing and clinically significant publications, including two original research article, one case report, and two narrative reviews. Considering the importance of shoulder function for daily activities, Wu et al. evaluated the cross-sectional area of the suprascapular nerves at multiple sites in different age and gender groups. They found that women had smaller sizes of the most proximal segment of the suprascapular nerves and the nerve cross-sectional area was larger in its distal division than the portion proximal to the mid-clavicular line. Their study is regarded as a fundamental reference for ultrasound imaging of diagnosing suprascapular neuropathy. Concerning the high prevalence of upper extremity nerve entrapment in the general population, Emril et al. conducted an observation study measuring the cross-sectional area of the median nerve in patients with carpal tunnel syndrome. There was high agreement between the diagnosis based on ultrasound imaging and that derived from electrophysiological findings. The research revealed the utility of ultrasound in diagnosing carpal tunnel syndrome in Indonesian population.

Furthermore, ultrasound facilities the exploration of the root causes of entrapment neuropathy. Cheng et al. reported two cases with carpal tunnel syndrome resulted from compression of solitary calcified nodules. Ultrasound prevented in one patient from unnecessary carpal ligament release. Karvelas and Walker reviewed the usefulness of ultrasound imaging for evaluation of distal ulnar nerve neuropathy. Terminal branches of the ulnar nerve like the dorsal and palmar ulnar cutaneous nerves had been elaborated in their article. Xiao and Cartwright provided a systematic protocol of scanning the radial nerve at the elbow level. The sonoanatomy and relevant clinical syndrome had also been described in their work.

As the responsible editors of the special issue, we are pleased to enlighten the utility of ultrasound in diagnosis of peripheral nerve disorders. Compared with conventional electric physiological testing, ultrasound assessment is less invasive and should be served as the first line for the evaluation of entrapment neuropathy. Furthermore, ultrasound imaging allows the investigators to simultaneously evaluate adjacent muscle echotexture and movement, making it the best imaging tool for serial follow-up after treatments. We also expect that the special issue will be served as a bridge to advanced research of ultrasound imaging in assessment and treatment of neuropathic pain syndromes.

## Author Contributions

K-VC has written the main text. All authors revised and approved the Editorial.

### Conflict of Interest

The authors declare that the research was conducted in the absence of any commercial or financial relationships that could be construed as a potential conflict of interest.
